# Effect of Clay Amendment and Strategic Deep Tillage on Soil Water Dynamics and Plant Growth Under Controlled Environments †

**DOI:** 10.3390/plants14050799

**Published:** 2025-03-04

**Authors:** Kanchana Wickramarachchi, Giacomo Betti, Gaus Azam

**Affiliations:** 1 Department of Primary Industries and Regional Development, Perth, WA 6000, Australia; kanch.wickramarachchi@dpird.wa.gov.au (K.W.); giacomo.betti@grdc.com.au (G.B.); 2 Grain Research and Development Corporation, Adelaide, SA 5065, Australia

**Keywords:** rainfed cropping, soil amelioration, root length density, topsoil water repellence, soil hydraulic conductivity, soil water capacity, soil evaporation, subsoil constraints

## Abstract

Strategic deep tillage (SDT) practices, such as soil mixing following the application of soil amendments, are promising approaches to alleviate topsoil water repellence and other subsoil constraints and improve crop productivity. However, there is a lack of knowledge on the effect of SDT on soil water dynamics, especially under water-limited environments. This study evaluates the effects of clay incorporation, soil inversion and deep soil mixing on soil water infiltration, surface evaporation rates, soil water storage and subsequent impacts on the below and aboveground growth of wheat (*Triticum aestivum* L. var Scepter) in controlled environments. Results show that soil mixing significantly improved water infiltration compared to an untreated control. Clay incorporation exhibited the highest bare soil surface evaporation rates immediately and two years post-tillage, leading to substantial water losses under warm and dry ambient conditions. Despite improving soil water storage in deeper layers, high evaporation rates in clay-incorporated soils negatively impacted wheat growth, with reduced shoot biomass and root length density. Conversely, soil inversion and mixing-only treatments demonstrated balanced improvements in water infiltration, soil water use, and wheat shoot biomass. These findings underscore the trade-offs associated with SDT practices, particularly in managing soil water loss and crop productivity in water-limited environments. This study also highlights the need for the careful selection of SDT for soil amelioration strategies tailored to soil types and climatic conditions to enhance agricultural productivity and sustainability.

## 1. Introduction

Soil constraints, particularly topsoil water repellence, subsurface acidity and compaction, are significant factors limiting crop productivity in agricultural systems worldwide [1,2,3]. On water-repellent soil, water tends to bead and run off instead of infiltrating the soil, leading to inefficient water use and poor crop establishment [4]. Acidic and compacted subsoils restrict root growth, limit access to water and nutrients and ultimately contribute to reduced crop yields [2,5,6]. Addressing these limitations is critical, especially for rainfed cropping systems in semi-arid and arid regions, where soil and water conservation play a central role in sustaining agricultural productivity. In recent decades, strategic deep tillage practices have gained attention in Australian broad acre farming as promising interventions to alleviate soil limitations by enhancing soil function and improving root access to deeper soil layers [5,6,7].

Strategic deep tillage (SDT), distinct from continuous or regular tillage, involves the occasional deep inversion, loosening, deep delving, mixing of soil layers or other physical disruptions to address specific issues such as topsoil water repellence, subsoil acidity and compaction [7,8,9,10]. This approach is particularly valuable in no-till or minimally tilled systems, where subsoil limitations can accumulate over time [9,11]. Research demonstrates that SDT can improve soil physical properties, enhance root penetration, and increase nutrient availability in deeper soil layers, collectively strengthening crop resilience in moisture-limited conditions [5,6,8,12,13].

In sandy-textured topsoils, the organic matter rich surface layers can also be water-repellent, which may affect soil water dynamics, such as infiltration and retention, in various ways [14,15]. Topsoil water repellence can increase runoff and typically results in preferential flow [14], but it can also act as an effective mulch, reducing loss of water from the subsurface layers by minimising evaporation and capillary rise [16]. SDT can be used to bury and dilute water-repellent topsoil, resulting in faster and more even water infiltration. However, this process may also eliminate the surface mulching benefits and increase evaporative water loss [16]. In furrow-sown cropping systems, water-repellent topsoil in the ridges can help channel water into the furrows [16,17]. This advantage is particularly beneficial in arid and semi-arid regions where rainfall is often low and irregular [18]. By harvesting water effectively, this approach can enhance early crop growth and nutrient uptake in furrow sown crops [19].

Clay spreading, or clay delving, involves incorporating clay-rich subsoil into the water-repellent sandy topsoils, and can reduce preferential water flow (finger flow) and result in deeper and more uniform wetting of the A horizon, particularly under initially dry conditions [10,13,15]. A long-term study involving multiple experiments suggests that the amelioration of water-repellent soils with SDT can improve crop yields and provide reliable benefits across most repellent soils in Western Australia (WA) [20].

Subsoil acidity is a major limitation in regions with sandy textured soils with low buffering capacity and high leaching, where low pH and high concentrations of aluminium (Al) ions restrict root elongation and access to subsoil moisture and nutrients, thereby increasing the vulnerability of crops to drought stress [6]. SDT can alleviate these effects by incorporating lime directly into the subsoil, neutralising acidity, and reducing Al toxicity within the root zone, thus facilitating deeper and more extensive root growth [6,21]. Enhanced root exploration improves water and nutrient uptake, offering a substantial advantage in drought conditions [6]. The incorporation of lime into acidic subsoils can provide a more rapid and lasting effect on pH amelioration than surface applications alone, as surface-applied lime often remains within the topsoil due to limited downward movement [22].

Similarly, soil hard pans caused by compaction from machinery traffic or through natural settling and cementation are another constraint that restricts root growth and crop productivity [23,24]. Hard layers impede water infiltration, reduce aeration, and limit root penetration, creating a suboptimal environment for crop growth [2,6]. When performed under suitable soil moisture conditions, SDT can disrupt hard and compacted layers, enhancing porosity and improving water movement throughout the soil profile. This reduction in soil strength allows for increased water infiltration and enables roots to explore deeper soil layers, which is particularly beneficial in rainfed systems where water availability can be inconsistent [5,25].

Despite these benefits, SDT has potential downsides, especially in low-rainfall, high-temperature regions where moisture retention and conservation are crucial. One significant drawback is the potential for increased soil water evaporation, which can reduce the amount of soil water available to crops and can negatively impact yield potential [20,26], especially when a tillage operation is implemented at a shallower depth than recommended for water-limited environments [27]. In particular, in a drier and hotter season in southwestern Australia, the SDT of sandy-textured, low water-holding-capacity soils can facilitate more rapid capillary movement of water to the surface and enhance the soil’s susceptibility to evaporation [26,28,29]. However, this could be the opposite in cooler and wetter environments and soils with higher clay contents, where summer tillage of topsoil is used to reduce soil evaporation by disrupting continuous soil porosity, thereby decreasing hydraulic conductivity [30].

The increased evaporation associated with strategic tillage is especially concerning in arid and semi-arid environments where rainfall is scarce and natural evaporation rates are high. In sandy soils, which have inherently low water-holding capacity, the water loss following tillage can be substantial, as these soils are more prone to rapid drying due to their larger pore sizes and lower organic matter content [31]. Additionally, tillage-induced evaporation can disrupt soil water storage, making crops more susceptible to drought stress if rainfall events do not closely follow tillage operations [28]. This challenge highlights the importance of timing the strategic tillage to align with anticipated rainfall, thereby maximising water retention and minimising evaporative losses [32]. Given the complex trade-offs associated with strategic deep tillage and soil amelioration for reducing the impact of soil constraints on crop productivity while increasing the potential soil water loss through enhanced evaporation, further research is needed to refine its application, especially in semi-arid and arid regions. This study, in a series of laboratory and glasshouse-based experiments, evaluates the effects of strategic tillage and clay incorporation on soil water infiltration, surface evaporation rates in water-repellent sandy soils and subsequent soil water storage and use and growth of a wheat crop (*Triticum aestivum* L. var Scepter).

## 2. Results

### 2.1. Soil Water Infiltration

**Figure 1:**
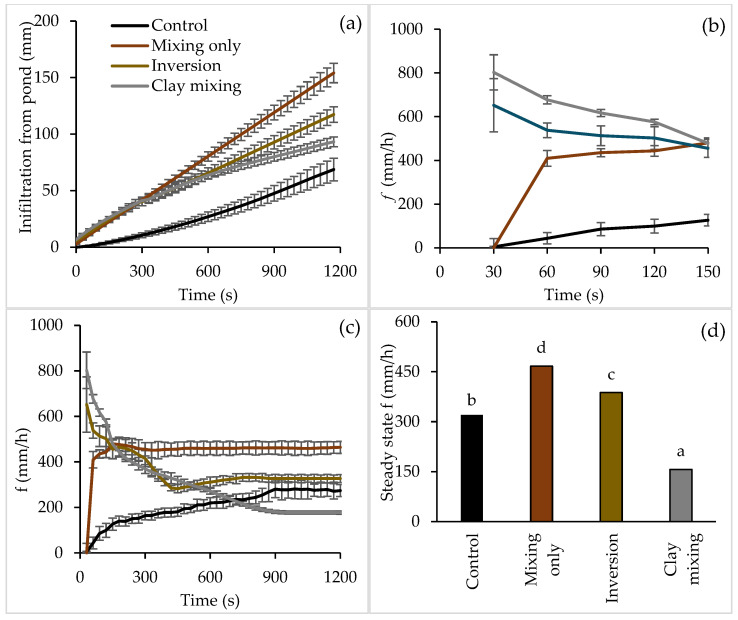
Infiltration of water (mm) from (**a**) the pond of the permeameter, (**b**) initial infiltration rate (*f*, mm/h), (**c**) infiltration rate (*f*, mm/h) over the duration of the experiment, and (**d**) steady state *f* (mm/h) for different soil amelioration treatments. Vertical error bars represent the standard error of the mean value. Bar graphs with different letters are significantly different at *p* ≤ 0.05. Note that the *Y*-axis scale differs between the figures.

The dynamics of water infiltration to reach a steady state varied among the four tillage treatments (Figure 1). However, all treatments reached a steady state within 20 min, as demonstrated in Figure 1. The cumulative infiltration of water from the pond over a period of 20 min was greater in all three treatments involving soil mixing and inversion compared to the untreated control (Figure 1a). The highest cumulative infiltration was recorded in the mixing-only treatment (154 mm), followed by soil inversion treatment (117 mm), then by clay incorporation (93 mm), and the lowest in the control (69 mm).

The water infiltration rate (f) exhibited a distinct temporal pattern across the different tillage treatments (Figure 1b,c). Initially, clay incorporation had the highest *f*, which gradually decreased over time (Figure 1b). The *f* reached a steady state in about 17 min in the clay incorporation treatment (Figure 1c). Soil inversion treatment had the second highest *f* at the beginning (Figure 1b), which decreased over time and reached a steady state in 10 min (Figure 1c). For both mixing-only and the control, the *f* rate increased over time. In soil mixing, the treatment took only 5 min to reach the steady state, whereas the control took 15 min to reach a steady state (Figure 1b,c).

The steady-state infiltration rates (*f*) were different across the tillage treatments (*p* ≤ 0.05), as shown in Figure 1d. Clay incorporation nearly halved the f (157 mm/h) compared to the control (320 mm/h). The inversion treatment increased the f by 21% (388 mm/h) compared to the control. Soil mixing treatment also increased the f by 46% (467 mm/h) compared to the control.

### 2.2. Evaporation from Bare Soil

**Figure 2:**
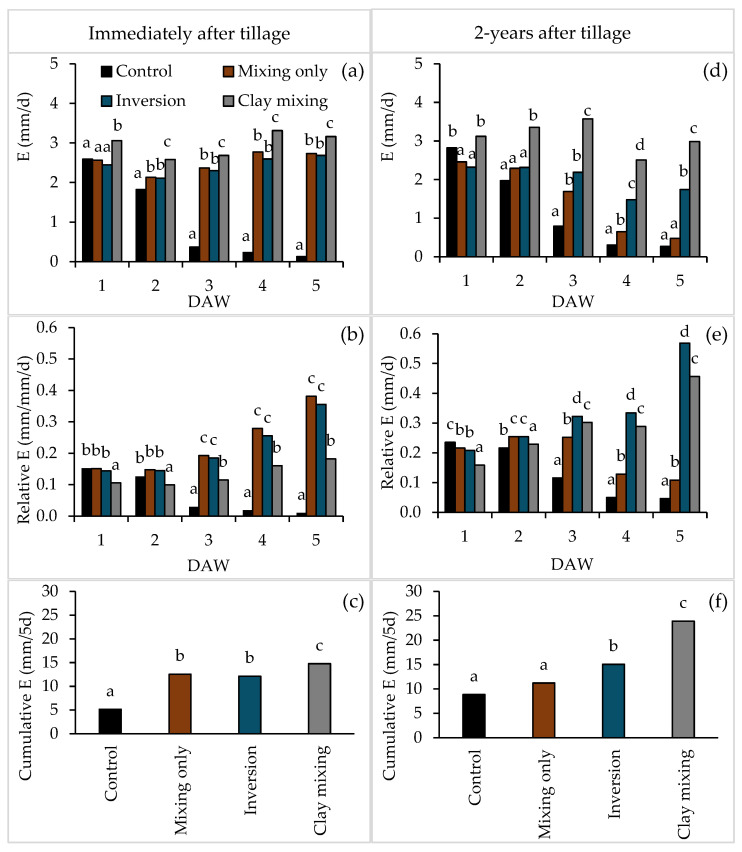
Daily loss of water through evaporation (E) from bare soil (**a**–**c**) immediately after tillage in 2019 at 30 °C and 80% relative humidity in a growth chamber and (**d**–**f**) two years after tillage in 2021 at 30 °C and 45% relative humidity in a glasshouse. DAW = days after watering. Vertical error bars represent the standard error of the mean value. Bar graphs with different letters for a given DAW are significantly different at *p* ≤ 0.05 (**a**,**b**,**d**,**e**). Note that the *Y*-axis scale differs between the figures.

Evaporation loss from the soil surface varied depending on the tillage treatment and time elapsed since tillage (Figure 2). In the growth chamber experiment conducted immediately following the construction of soil columns, which is comparable to the first few days of field conditions following tillage treatments, the evaporation rate for clay incorporation was consistently higher at every measurement compared to the other three treatments over the 5-day measurement period (Figure 2a). On day 1, there was no difference among the three treatments that did not include clay incorporation. However, during the next 4 days, mixing-only and inversion treatments had higher evaporation rates compared to the control (Figure 2a).

The relative evaporation rate (mm water loss per mm of soil water per day) also varied between the tillage treatments (Figure 2b). On day 1, the clay incorporation treatment exhibited a lower relative evaporation rate compared to the three treatments without clay incorporation. Furthermore, the relative evaporation rate of the clay incorporation treatment remained consistently stable throughout the 5-day measurement period. Over the next four days, both mixing-only and inversion treatments showed a consistent increase in relative evaporation rates, which were higher compared to the control and clay incorporation. Conversely, the relative evaporation rate of the control decreased rapidly from day 2 and remained very low until measurements ceased. The cumulative evaporation in the control, over five days, was lower than other treatments (Figure 2c). Mixing-only and inversion treatments had lower cumulative evaporation than clay incorporation (Figure 2c).

Two years post-tillage, under glasshouse conditions (Figure 2d–f), the evaporation rate of clay incorporation was higher from day 2 onwards compared to the other three treatments (Figure 2d). The daily difference in evaporation and relative evaporation rates between the control and the mixing-only treatments was generally less pronounced than what was observed two years earlier (Figure 2d,e). After saturation, clay incorporation had consistently higher daily evaporation rates over the 5-day period (Figure 2d), leading to greater cumulative water loss (Figure 2d). The cumulative evaporation in control and mixing-only treatments over 5 days were lower than the inversion and clay incorporation treatments (Figure 2f), with clay incorporation again having the highest cumulative evaporation among all the treatments, consistent with the results observed 2 years prior.

### 2.3. Wheat Plant Growth

**Figure 3:**
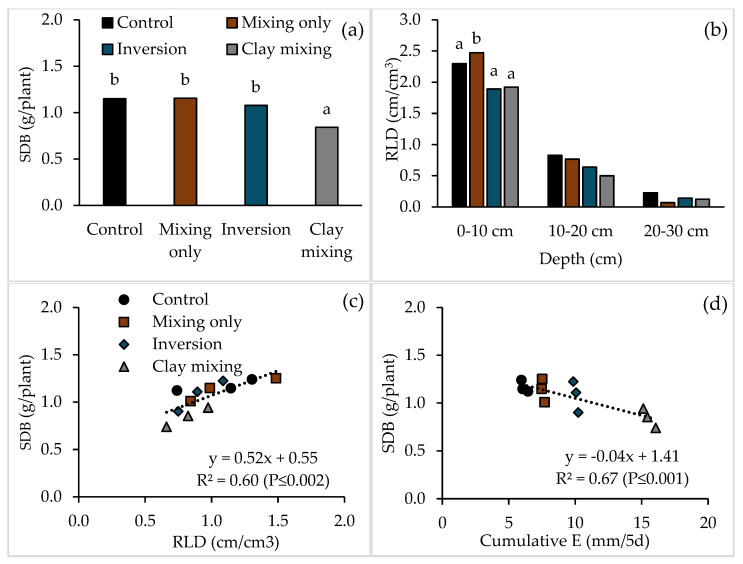
Effect of tillage treatments on (**a**) shoot dry biomass (SDB) and (**b**) root length density (RLD). Relationship between the wheat (*Triticum aestivum* L. var Scepter) shoot dry biomass and (**c**) total RLD, 0–30 cm, and (**d**) cumulative evaporative loss from the bare soils. Bar graphs with different letters are significantly different at *p* ≤ 0.05 and the absence of letters refers to non-significance (for 10–20 and 20–30 cm depths) at *p* ≤ 0.05. Note that the *Y*-axis scale differs between the figures.

Wheat shoot dry biomass varied among treatments, where clay incorporation had the lowest shoot dry biomass compared to other treatments (Figure 3a). The root length density (RLD), calculated as the total length of a root in a unit volume of soil, also varied among the treatments (Figure 3b). In the topsoil (0–10 cm), soil mixing-only had greater RLD compared to inversion and clay incorporation treatments. The RLD in the control was not different from any other treatment. Generally, the subsoil (10–30 cm) had a smaller RLD than the topsoil, and there was no difference in RLD between the treatments.

Root growth was significantly correlated (*p* ≤ 0.002) with shoot dry biomass across treatments (Figure 3c). The relationship between RLD and shoot biomass was positive, i.e., soil columns with greater RLD also had greater shoot dry biomass. The cumulative evaporation from the bare soils (Figure 3d) was also significantly correlated (*p* ≤ 0.001) with shoot dry biomass, and the relationship was negative (Figure 3d). Clay incorporation had the highest cumulative evaporation and lowest shoot dry biomass (Figure 3d).

### 2.4. Soil Moisture Status During the Wheat Growing Experiment

**Figure 4:**
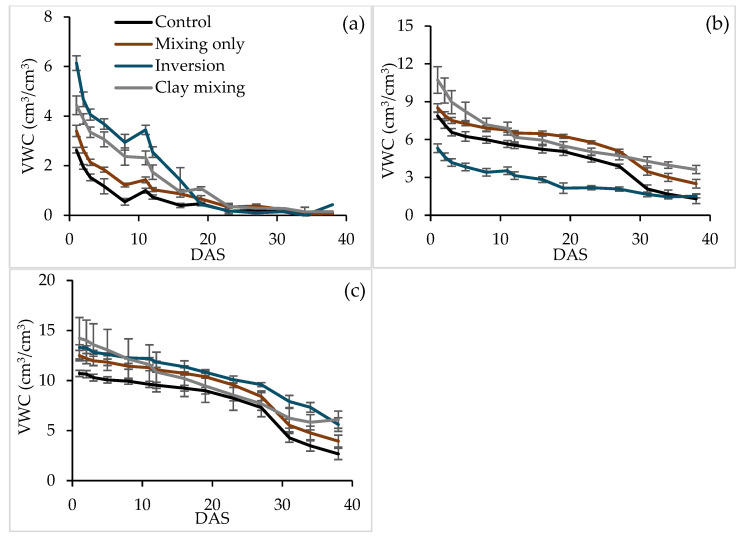
Soil volumetric water content (VWC, cm^3^/cm^3^) at (**a**) 0–10 cm, (**b**) 10–20 cm, and (**c**) 20–30 cm depths under four tillage treatments over 38 days after seeding (DAS). Vertical error bars represent the standard error of the mean value. Note that the *Y*-axis scale differs between the figures.

Soil volumetric water content (VWC) at different depths over time followed a distinct pattern between the tillage treatments (Figure 4). During the first few measurements, VWC at 0–10 cm varied between the tillage treatments in the order of inversion > clay incorporation > mixing-only > control (Figure 4a). Treatments with higher VWC in the 0–10 cm soil layer experienced more rapid depletion of the water content in this layer. At around 20 DAS and thereafter, there was no difference between treatments in VWC at 0–10 cm (Figure 4a). At 10–20 cm depth, VWC also varied between the treatments. At this depth, the inversion treatment, with the original water-repellent topsoil buried in this layer, had the lowest VWC (Figure 4b). From day 31 onwards, there was no difference between the control and the inversion treatments in VWC status at 10–20 cm (Figure 4b). During these later measurements, both soil mixing treatments had higher VWC than the other two treatments. Clay incorporation had a higher VWC than the mixing-only treatment after day 30. At a 20–30 cm depth, a similar pattern in VWC was observed in all treatments (Figure 4c). Overall, evapotranspiration losses were highest in the clay incorporation treatment and lowest in the control.

## 3. Discussion

This study investigates the effects of various tillage treatments on soil water infiltration, evaporation, and plant growth, providing valuable insights into the interactions between soil management practices, water dynamics, and crop performance. The findings reveal significant differences among treatments, with implications for water conservation and sustainable agricultural practices.

### 3.1. Soil Water Infiltration Dynamics

The water infiltration dynamics varied among the four tillage treatments, with the soil mixing-only treatment having the highest cumulative infiltration and steady-state infiltration rates, followed by inversion tillage, clay incorporation, and the control (Figure 1). The control with water-repellent topsoil initially exhibited a near-zero infiltration rate (Figure 1b) and consistently showed the lowest infiltration compared to all tillage treatments. The infiltration rate of control increased over time, aligning with the infiltration patterns observed in water-repellent soils, as reported by Feng et al. [33] and Wang et al. [34]. In the control treatment, water repellency of the top layer and layered non-uniform hydraulic conductivity might have created an unstable wetting front with preferential flow paths [15,35,36].

The highest cumulative infiltration and steady-state infiltration rates were observed in the soil mixing-only treatment, highlighting the benefits of soil mixing to treat water-repellent surface soil, enhance soil porosity and hydraulic conductivity [36,37] and improve crop productivity [20]. The higher infiltration rate observed with soil mixing, compared to the control, may result from the dilution of repellent sand with non-repellent sand from the subsurface during the mixing process [15]. Further, uniform soil texture with uniform hydraulic conductivity obtained through soil mixing resulted in a stable wetting front compared to the control [35]. This is apparent in the shorter time taken to achieve the stable state compared to the control.

Both soil inversion and clay incorporation exhibited initially high infiltration rates, which gradually declined before reaching a steady state. This is a typical pattern for wettable soils [34]. In the inversion treatment, bringing the wettable sand to the surface while burying the water-repellent soil at a 10–20 cm layer created a non-uniform, layered hydraulic conductivity change down the soil profile. However, a wettable layer over the underlying water-repellent layer may not have resulted in non-uniform soil wetting, as reported by previous researchers [38,39]. A more persistent decline in infiltration rate in clay incorporation reflected the sealing effects of fine clay particles on pore spaces. The findings related to clay incorporation aligned with Assouline [40], who demonstrated the trade-offs of clay amendments in altering soil hydraulic conductivity. A higher steady-state infiltration rate underscores the advantages of soil tillage treatments such as mixing-only and inversion in maintaining water recharge and reducing surface runoff, critical for large rainfall events in rainfed cropping systems, while an initial higher infiltration rate followed by a moderate steady state infiltration rate, as observed in clay incorporation, has the potential to maintain quick recharging of rainfall and hold more water in the root zone for a longer time [30,41].

### 3.2. Evaporation Dynhamics from Bare Soil

Evaporation dynamics immediately after tillage (growth chamber conditions) and two years later (glasshouse conditions) revealed persistent differences among treatments. Clay incorporation consistently resulted in the highest evaporation rates across both timeframes, highlighting its susceptibility to surface water loss due to increased water holding near the soil surface, leading to enhanced capillary uplift [40,42].

In contrast, the control treatment exhibited the lowest evaporation rates, likely due to the undisturbed water-repellent soil surface limiting water movement to the liquid–gas phase of evaporation and vapour loss. It has been suggested previously that soil layers exhibiting water-repellent properties can mitigate evaporative loss by impeding vapour flow and diffusion [16]. This phenomenon is attributed to the modified geometry of the liquid/gas interface in partially saturated regions [43,44] and the diminished capillary rise needed to replenish the upper soil layer due to the presence of a water-repellent layer [43,45]. Laboratory studies provide some evidence of reduced evaporation losses in uniformly wetted hydrophobic soils compared to wettable soils. Even a thin layer of water-repellent material can decrease evaporation, with an increased depth of the hydrophobic layer leading to a greater reduction [46]. Mixing-only and inversion treatments exhibited intermediate evaporation rates, higher than those of the control but lower than those observed with clay incorporation [47]. These patterns align with findings by Unger and Cassel [48], emphasising the role of moderate tillage in balancing soil water retention and surface loss.

Two years post-tillage, the effects of clay incorporation on evaporation persisted, underscoring the long-term implications of soil structure modifications. While inversion treatments showed increased evaporation relative to the control, mixing-only treatments demonstrated more sustainable evaporation rates, likely due to gradual soil stabilisation and organic matter accumulation over time [49].

### 3.3. Soil Water Use and Plant Growth

**Figure 5:**
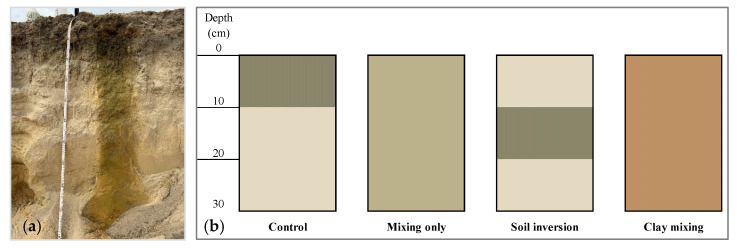
(**a**) A typical sandy soil profile in southwestern Australia and (**b**) a schematic of the four tillage treatments.

The soil VWC varied with depth and time among treatments. In the topsoil (0–10 cm), the inversion treatment initially retained the highest VWC, but this advantage diminished after 20 days as water was rapidly depleted (Figure 5a). Clay incorporation and mixing-only treatments exhibited higher VWC at greater depths (10–30 cm) compared to the control and inversion treatments, reflecting improved water redistribution following soil disturbance [15,48].

The effects of tillage treatments on wheat growth, including shoot dry biomass and RLD, closely mirrored the patterns of soil water status and water infiltration. Mixing-only and inversion treatments supported greater root proliferation and shoot dry biomass compared to the control and clay incorporation treatments. These results align with the findings of other researchers [50], who noted that soil disturbance practices can improve root growth by reducing compaction and enhancing water and nutrient access.

However, clay incorporation, despite its potential to improve infiltration, consistently resulted in the lowest shoot dry biomass due to high evaporation rates and surface water losses and potentially due to a higher wilting point, leading to a significant reduction in plant available water. This highlights the need to pair clay incorporation with complementary practices such as residue retention or mulching to mitigate evaporative losses and enhance water use efficiency [48,51] or to evaluate whether the practice of clay incorporation is necessary in a water-limited environment where evaporation losses far exceed precipitation, especially in the drier season [26].

### 3.4. Integrating Tillage Practices for Sustainable Agriculture

The relationships between infiltration, evaporation, and plant growth underscore the complexity of selecting tillage practices for sustainable agricultural systems. Soil mixing-only and inversion treatments have emerged as promising strategies for improving infiltration and plant growth, particularly in water-limited environments. However, their effectiveness may depend on integrating these practices with measures to minimise evaporation and enhance soil organic matter content over time [51].

Clay incorporation, while beneficial for addressing topsoil water repellence, poses significant challenges related to higher evaporative losses and soil surface sealing. Implementing complementary strategies, such as surface mulching, cover cropping, or limiting the frequency of interventions, could enhance its sustainability [51]. The control treatment, characterised by minimal disturbance, demonstrated superior water conservation but limited infiltration and crop growth benefits. This highlights the trade-offs associated with reduced tillage and the importance of context-specific decision-making in soil management using strategic tillage [8,20].

## 4. Materials and Methods

### 4.1. Soil Collection and the Preparation of Columns

In March 2019, bulked sandy topsoil (0–10 cm depth) and subsoil (20–40 cm depth) were collected from a continuously cropped field (hereafter referred to as paddock) near Moora, WA (−30.756029, 115.849207). The soil at the site is classified as Yellow Arenosol in the Australian Soil Classification [52]. A bulked medium clay subsoil was collected from a nearby paddock (−30.772631, 115.857166) for clay incorporation treatment. All soils were air-dried separately at 40 °C before they were tested for physical and chemical properties. The physical and chemical properties of the sand and clay are also presented in Table 1. The topsoil was severely water-repellent, with a value of molarity of ethanol droplet (MED) of 2.4, and the subsoil soil was wettable with a MED value of zero [53]. The paddock had been previously treated with agricultural lime, resulting in soil pH levels above the critical values of 5.5 in the topsoil and 4.8 in the subsoil [54]. Generally, the soil had low chemical fertility with a topsoil organic carbon (OC) content of 0.71%, and the essential nutrient contents were at low levels.

Air-dried soils were packed into PVC columns to a height of 30 cm to establish the following four SDT-related soil amelioration treatments: (i) untreated control (0–10 cm topsoil and 10–30 cm subsoil), (ii) mixing-only: sandy topsoil and subsoil (one part topsoil mixed with two parts subsoil) to represent soil mixing tillage to a 30 cm depth operation such as rotary spading [7], (iii) soil inversion: 0–10 cm subsoil, 10–20 cm topsoil and 20–30 cm subsoil to represent the full soil inversion profile created following mouldboard ploughing, and (iv) clay incorporation into topsoil and subsoil: (one part topsoil: two parts subsoil, mixed with clay-rich subsoil at 200 t clay-rich subsoil/ha equivalent) to represent clay spreading followed by rotary spading to 30 cm depth (Figure 5). All treatments were replicated four times. The soil mixing to create treatments (i) and (vi) was achieved using a cement mixer (Ozito brand, Model CMX120, Ozito, Australia). Mixed soils were packed into polyvinyl chloride (PVC) columns 35 cm tall with an inner diameter of 28 cm. Soils were packed carefully, from the bottom to the top at 10 cm depth increments, avoiding any gaps between soil and the PVC pot wall, to an optimum bulk density of 1.5 g/cm^3^. The outer wall of the PVC columns was wrapped with an aluminium-based insulation material to minimise temperature fluctuation. The base of the PVC columns was closed with PVC end caps, and 10 holes (3 mm diameter) were drilled in the base to facilitate drainage during the infiltration experiment and to avoid any potential perched waterlogging during plant growth.

### 4.2. Experiment 1—Measurements of Hydraulic Conductivity

**Figure 6:**
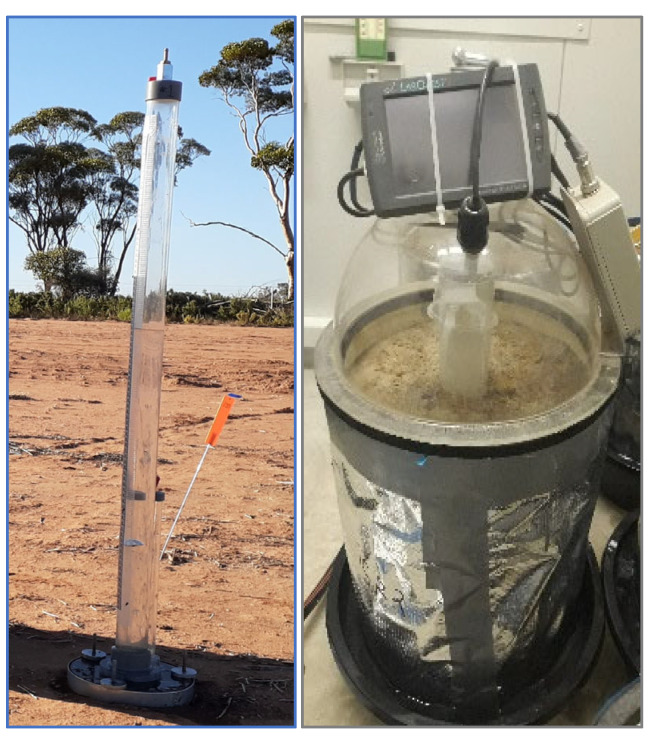
(**left**) CSIRO disc permeameter, and (**right**) a soil column with the evaporation dome.

Air-dried soil columns were used to measure near-saturated and steady state hydraulic conductivity (hereafter infiltration rate, *f*). A Commonwealth Scientific and Industrial Research Organisation (CSIRO) disc permeameter [59] was used to measure hydraulic conductivity (Figure 6, left). Philip’s equation [60] was used to calculate the *f* rates. All columns were arranged in a randomised block design under shade at approximately 25 °C temperature. Rainwater was used for the *f* to represent the rainfed cropping system in WA.

### 4.3. Experiment 2—Evaporation from Bare Soils

Soil water loss through evaporation (E) from bare soil was measured in 2019 and 2021 under two controlled environmental conditions. In 2019, when f measurements were completed (Experiment 1), soil columns were covered for 48 h to allow free drainage of excess water and field capacity to be reached. The soil columns were subsequently transferred to a growth chamber, where they were arranged following the same randomised block design. The growth room was equipped with artificial lighting (approximate light intensity of 400 µmol/m^2^/s) for 12 h a day and set to 30 °C and 40% relative humidity, simulating the warm and dry conditions typical of most crop-growing regions in southwestern WA. After completing evaporation measurements in 2019, all soil columns were stored outdoors and exposed to natural environmental conditions, allowing the soil to resettle similarly to how it occurs in the field after strategic tillage. The second set of measurements was taken in May 2021—two years after the construction of soil columns. The second set of measurements was taken in a controlled-environment glasshouse set at 30 °C temperature and natural relative humidity (around 40%) and natural light (around 11 h of daylight per day).

On both occasions, the same protocol was followed, where soil columns were wet up to near saturation before taking E measurements. The columns were saturated gradually by using a manual drip irrigation system to minimise soil recompaction caused by irrigation. A kitchen towel was placed on the soil surface during irrigation to avoid ponding and soil surface disturbance. Vapour pressures were measured using a custom-made rapid evaporation dome [61] equipped with a combined temperature and humidity sensor (HTM2500LF—Temperature and Relative Humidity Module, TE Connectivity, Berwyn, PA, USA) mounted inside the dome, which was interfaced with a LabQuest^®^ v2 data logger (Vernier Software & Technology, Beaverton, OR, USA). A small fan (operated by a motor with an electronic speed controller and propeller) was installed at the inner apex of the dome. Its purpose was to mix the air within the dome for uniform measurements and obtain a good response time for the sensor. The fan-generated wind speed did not exceed 0.3 m s^−1^. The inner diameter of the evaporation dome matched that of the PVC column. The rapid evaporation dome was positioned above the soil column and the foot of the dome created a seal with the upper rim of the PVC tube (Figure 6, right).

The LabQuest^®^ data logger collected 20 vapour pressure measurements per second for about 30 s (Figure 6, right). The data logger was pre-programmed to calculate Kelvin temperature, saturation vapour pressure, vapour pressure, and vapour density. It was configured to graph vapour density (g m^−2^) against time (minutes) during the real-time data collection. The resulting graph was used to capture the slope of the linear regression line (only the best linearly fitted data points were used), which was expressed in g m^−2^ min^−1^. This value was subsequently converted to instantaneous evaporation (E) mm/day. Four vapour measurements were taken from each column daily, at hourly intervals between 10 a.m. and 2 p.m., and an average evaporation rate was calculated from the four measurements at each measurement date. The vapour measurements were repeated over five consecutive days after saturation. Soil columns were left open to evaporate naturally without any further water during the 5-day period of the evaporation measurements.

### 4.4. Experiment 3—Measurement of the Wheat Growth and Soil Water Status

Once evaporation measurements were completed, a PVC moisture access tube was installed at the centre of each soil column to measure soil water status in the column using a DIVINER 2000 capacitance probe (Sentek Sensor Technologies, South Australia, Australia). Soil columns were moved to a controlled-environment glasshouse and arranged using the same randomised block design. The glasshouse temperature was set at 25 °C. Soil columns were rewatered to near saturation and left to drain for 48 h until they reached a drained upper limit, when each soil column was weighed. Each column was planted with 30 wheat (*Triticum aestivum* L. var Scepter, Australian Grain Technologies, South Australia, Australia) seeds at 3 cm depth. Each column was fertilised uniformly with 37 kg/ha of mono-ammonium phosphate (21.9% phosphorus, 10% nitrogen) and 57 kg/ha of urea (46% nitrogen) in each season. Mono-ammonium phosphate was applied at sowing 3 cm below the seed, and urea was surface-applied at the Z14 [62] growth stage for wheat to simulate typical field fertilisation techniques. Eight days after seeding (DAS), plant emergence was counted, and extra plants were removed to retain only 10 plants per pot (140 plants/m^2^). From this day onward, the columns were irrigated once a week using manual drip irrigation. The amount of water added was equal to the water lost due to evapotranspiration during the previous period. Prior to any irrigation, soil moisture status was also measured at 10 cm depth intervals to a depth of 30 cm using the DIVINER 2000 capacitance probe. The Diviner 2000 probe was calibrated for this soil type by measuring bulk density and gravimetric water content [6].

Wheat plants were harvested at 40 DAS when the crop was at the Z24-26 growth stage [62]. Wheat shoots were clipped off at the soil surface, dried at 60 °C and weighed to determine shoot dry biomass. Then, the soil from the soil column was recovered separately into three segments (0–10, 10–20, and 20–30 cm), starting from the surface. Roots in each segment were collected by gently washing the soil through a 4 mm sieve using a gentle jet of water. Roots from each depth segment were washed, scanned and analysed with the commercial software package WINRHIZO™ Pro 2007a (Regent Instruments, Québec City, QC, Canada) for total root length, average diameter, and volume.

### 4.5. Statistical Analyses

All statistical analyses were conducted using GenStat statistical software (Version 22, VSN International, Oxford, UK). A one-way analysis of variance (ANOVA) was performed to evaluate the effects of treatments on steady state infiltration, evaporation, relative evaporation, cumulative evaporation, shoot weight and root length density. Fisher’s protected least significant difference (LSD) test was applied at a significance level of *p* ≤ 0.05 to identify significant differences between treatment means. Finally, regression analyses were performed to assess the strength and direction of linear relationships between RLD and shoot weight, as well as cumulative evaporation and shoot weight. In each regression analysis, the R-squared (R2) value was reported to evaluate the strength and direction of the relationships, while a probability (*p*) value was provided to indicate the significance of the relationship among the parameters. A relationship was considered significant at *p* ≤ 0.05.

## 5. Conclusions

This study investigates the impacts of strategic tillage and soil amendments on soil water dynamics and crop productivity. The findings indicate a trade-off between enhanced water infiltration and increased evaporative loss resulting from soil mixing and inversion. Although clay incorporation initially improves soil structure and water availability, it subsequently leads to elevated evaporation rates and reduced crop biomass under hot and dry environments. However, previous research [10,13] suggests that the extent of soil mixture significantly influences these outcomes. It is essential to further explore these effects to optimise farming practices associated with strategic tillage.

To enhance the understanding and implementation of strategic tillage practices, future research should examine the combined effects of strategic tillage with techniques for reducing evaporative losses (such as surface mulching, cover cropping, and residue retention) and increasing water use efficiency. Long-term field experiments are necessary to assess the sustainability and cumulative effects of strategic tillage practices on soil water dynamics, crop productivity, and soil condition. Such studies should encompass diverse climatic conditions and soil types to provide comprehensive insights. It is crucial to continue this research in the field, where soil profiles after strategic deep tillage are more complex and variable than those reconstructed in laboratory conditions.

In conclusion, while strategic tillage and soil amendments offer promising solutions to overcome soil constraints and improve crop productivity, a holistic and site-specific approach is essential to address the associated trade-offs. By integrating complementary practices, conducting long-term studies, and leveraging advanced monitoring techniques, we can enhance the sustainability and resilience of agricultural systems in water-limited environments.

## Figures and Tables

**Table 1:** Physical and chemical properties of the topsoil, subsoil and the clay used in the experiments. Note: ND = not detected.

Soil	Textural Grade (Clay Content, %) [55]	Molarity of Ethanol Droplet (MED) Score [53]	Organic Carbon (%) [56]	Total Inorganic Nitrogen (mg/kg) [57]	Colwell Phosphorus (mg/kg) [58]	Colwell Potassium (mg/kg) [58]	Sulphur (mg/kg) [57]	pH in 0.01 M CaCl_2_ [57]	Electrical Conductivity (dS/m) [57]
Topsoil	Sand (3%)	2.4	0.71	24.7	25.7	20.5	5.4	6.2	0.06
Subsoil	Sand (4%)	0	0.16	2.5	8.2	ND	4.1	4.8	0.01
Clay rich subsoil	Sandy clay (38%)	0	0.36	26.7	20.0	28.2	129.7	6.1	0.49

## Data Availability

The datasets used and/or analysed during the current study are available from the corresponding author upon reasonable request.
